# In-Depth Analysis of Physiologically Based Pharmacokinetic (PBPK) Modeling Utilization in Different Application Fields Using Text Mining Tools

**DOI:** 10.3390/pharmaceutics15010107

**Published:** 2022-12-28

**Authors:** Aleksandra Krstevska, Jelena Đuriš, Svetlana Ibrić, Sandra Cvijić

**Affiliations:** 1 Department of Pharmaceutical Technology and Cosmetology, University of Belgrade—Faculty of Pharmacy, Vojvode Stepe 450, 11221 Belgrade, Serbia; 2 Alkaloid AD Skopje, Blvd. Aleksandar Makedonski 12, 1000 Skopje, North Macedonia

**Keywords:** physiologically based pharmacokinetic modeling (PBPK), physiologically based biopharmaceutics modeling (PBBM), text mining, topic modeling, modeling and simulations (M&S)

## Abstract

In the past decade, only a small number of papers have elaborated on the application of physiologically based pharmacokinetic (PBPK) modeling across different areas. In this review, an in-depth analysis of the distribution of PBPK modeling in relation to its application in various research topics and model validation was conducted by text mining tools. Orange 3.32.0, an open-source data mining program was used for text mining. PubMed was used for data retrieval, and the collected articles were analyzed by several widgets. A total of 2699 articles related to PBPK modeling met the predefined criteria. The number of publications per year has been rising steadily. Regarding the application areas, the results revealed that 26% of the publications described the use of PBPK modeling in early drug development, risk assessment and toxicity assessment, followed by absorption/formulation modeling (25%), prediction of drug-disease interactions (20%), drug-drug interactions (DDIs) (17%) and pediatric drug development (12%). Furthermore, the analysis showed that only 12% of the publications mentioned model validation, of which 51% referred to literature-based validation and 26% to experimentally validated models. The obtained results present a valuable review of the state-of-the-art regarding PBPK modeling applications in drug discovery and development and related fields.

## 1. Introduction

Modeling and simulation (M&S) is a proven scientific approach that has a profound impact on drug discovery and development processes. Physiologically based pharmacokinetic (PBPK) models represent mechanistic models, which integrate a series of different mathematical equations describing the processes a drug undergoes in a human (or animal) organism. Since PBPK models are based on known anatomical and physiological data, they are very useful for the prediction of pharmacokinetic (PK) and pharmacokinetic-dynamic (PK/PD) behavior of drugs and/or chemicals. The term PBPK modeling dates back to 1937 when Teorell introduced mathematical equations to describe drug concentrations over time in blood and body tissues [1]. However, the interest in PBPK modeling increased during the early 1970s due to the increased availability of computers and numerical integration algorithms [2,3]. The expansion of interest in PBPK modeling has been perceived through the growth in the number of scientific publications that include PBPK modeling in the past decade [3,4]. PBPK modeling finds application in every phase of the drug development process, starting from the early drug discovery phase where limited data are available, continuing all the way to the late stages of drug development. The major advantage of PBPK modeling over other modeling tools used in early drug discovery stages is that PBPK models provide a holistic view of the interplay between different parameters on drugs’ PK behavior [5]. Therefore, publications that include the use of PBPK modeling to inform early drug discovery and development are constantly increasing [5,6,7,8,9,10,11,12]. The application of PBPK modeling is even wider during the clinical development phase, where it can be applied in numerous areas such as the simulation/prediction of drugs’ PK in different populations, e.g., pediatric [13,14,15,16,17], elderly [18,19,20,21], pregnant women [22,23,24] etc. It can also be used for the prediction of changes in the intrinsic physiological factors, such as organ impairment on a drug’s PK [25,26,27,28], drug-drug interactions (DDIs) [29,30,31], oral drugs absorption and formulation effects on drug absorption and disposition [32,33,34,35]. This constant increase in the number of publications related to PBPK modeling can be attributed to a variety of factors, including the increased availability of user-friendly PBPK dedicated platforms, which have largely facilitated the use of PBPK modeling tools, even by users with limited modeling experience [36]. Additionally, some of the platforms such as GastroPlus^TM^ and PK-Sim^®^ have changed their course from being commercial to open access platforms [37]. However, the rise in popularity of these easy-to-access in silico tools often leads to their inappropriate use by non-sufficiently trained users with the sole aim of complementing their scientific publications, increasing the risk of generating meaningless and false predictions [38]. This issue is associated with another challenge, i.e., determination of the proper procedure to assess model validity. Until recently, neither the FDA nor EMA provided guidance on PBPK model validity assessment during the regulatory review process. In 2018, both the FDA and EMA released the first guidelines on how to report and qualify PBPK models intended to support a regulatory decision [39,40]. However, these recommendations are rarely followed in research studies published in peer-reviewed journals [41].

In the past 10 years, a large number of review articles on PBPK modeling have been published; however, only a small number of papers focus on the application of PBPK modeling across different areas. Some of them cover only a specific therapeutic area [42,43,44,45,46,47], while others review the utilization of PBPK models from a regulatory submission perspective or industry perspective [48,49,50]. One recent review paper analyzed the use of PBPK models over a variety of fields but focused mainly on the applications related to drug development, excluding the articles exclusive to toxicology and environment risk assessment (ERA) [37].

Due to a large number of publications on PBPK modeling and considering the versatility of applications, the traditional literature review on this topic would be quite time-consuming. Computational text mining tools, on the other hand, are advantageous because a large set of textual data can be analyzed in a short period of time. Text mining uses natural language processing (NLP) to transform the unstructured text in documents and databases into normalized, structured data suitable for analysis [51]. Text mining identifies facts, relationships and assertions that would otherwise remain hidden in the mass of textual big data. Topic modeling is a text analysis method capable of scanning a set of documents, detecting word and phrase patterns within them and automatically clustering word groups and similar expressions that best characterize a set of documents. The literature shows that in the past few years, text mining tools have also been used in biomedical research [52,53,54,55].

The aim of this review was to use text mining tools to conduct an in-depth analysis of literature sources on PBPK modeling published in the last 10 years in relation to the application fields of these models in different therapeutic areas, target species, routes of administration etc. Furthermore, the analysis addressed the proportion of publications that include model validation and distribution of different modeling software packages across the extracted publications.

## 2. Methods

### 2.1. Data Sources and Search Method

Text mining of the literature was performed using Orange 3.32.0 software [56], an open-source data mining platform. Using the Add-ons option, the add-on for text mining was installed. For the purposes of this review, data was fetched using the PubMed widget, an integral part of the text mining section that allows search and retrieval of information from the PubMed website. The following search keywords were inserted in the advanced search option: “PBPK” [Title/Abstract] OR “Physiologically based pharmacokinetic” [Title/Abstract] OR “Physiologically based modeling” [Title/Abstract] OR “Physiologically based model” [Title/Abstract] OR “Physiologically based pharmacokinetic-dynamic modeling” [Title/Abstract] OR “Physiologically based biopharmaceutics modeling” [Title/Abstract] OR “PBBM” [Title/Abstract] OR “Model informed drug development” [Title/Abstract]. The filter was set to only retrieve the papers published in the period between 01/01/2012 and 18/05/2022. All the collected papers were downloaded for further analysis, but to facilitate the construction of the tokens, only the article abstract was kept as a meta-feature. The downloaded files were incorporated in the corpus widget. For visualization of the abstract, title of publication and date of publication, the corpus viewer widget was used.

### 2.2. Eligibility Criteria

According to the selected setup, each article included in the text mining analysis had to meet the following criteria: (1) to be published in English, (2) to have abstract available for analysis, (3) to be published between 2012 and 2022, (4) to contain at least one of the selected keywords either in the title, abstract or keywords, and (5) to present either an original research article, white paper, review article, perspective, book or supplement articles. This last criterion included reviewed pre-prints published online ahead of print. Other types of manuscripts such as tutorials, conference papers, workshops, commentaries, editorials, protocols, pre-prints that precede a formal review process etc. were excluded from further analysis.

### 2.3. Text Preprocessing

Several pre-processing steps were applied to transform the textual data into numerical features that can subsequently be used for further analysis. The preprocessing of text helps to split the raw data into smaller tokens (words or units) and understand the data. Four methods were used to preprocess the data: transformation, tokenization, filtration and and-gram ranging. Within the transformation method, the lowercase option was selected, which transforms all words into lowercase. Additionally, URLs were removed from the text. Secondly, with the help of the tokenization method, the text was split into smaller elements (words, sentences, phrases, parts of speech and n-grams). The “regular expression” (Regexp) technique was selected to omit punctuations from the data. The expression pattern was used to single out only those words that have more than three letters. Next, the data were filtered by removing the so-called “stop words” that are commonly used words in a given language but have no particular meaning. For this purpose, a text file containing the list of the most commonly used “stop words” including prepositions, pronouns, conjunctions etc. was created. The list of “stop words” was further supplemented with words that are not relevant to the analysis and often occur within the text such as: “pbpk”, “model”, “based”, “physiologically”, “pharmacokinetic”, “modeling”. Additionally, filtering out the numbers option was selected for filtering out whole numbers from the text. The last input in the filtration method was the selection of the 100 (default value) most frequent tokens. Afterwards, the option WordNet Lemmatizer was chosen for stemming and lemmatization to words. Lastly, the default value of one and two grams was kept for the n-gram range.

### 2.4. Widgets to Determine Word Counts, Frequency and Significance

The word cloud widget was used to visually display the most frequently mentioned words (tokens) in the text, where the size of the word is proportional to the frequency of their mention in the text. This widget outputs a list of words sorted by their frequency. Statistically significant words for the entire corpus, as well as for each topic, were identified by connecting the bag of words widget with the word enrichment widget and corpus viewer tool. The significance of each word was determined based on the *p*-value. The *p*-value was set to 0.05, meaning that words with a *p*-value lower than 0.05 are significant for a selected subset compared to the entire corpus. The most relevant keywords were identified using the extract keyword widget, and the word importance was calculated with the “term frequency-inverse document frequency” (TF-IDF) method. The TF-IDF method computes the frequency of each term in the document and weights it by the opposite of the term’s frequency in the whole corpus. The terms with the highest scores were submitted to further analysis.

### 2.5. Topic Modeling

Topic modeling discovers abstract topics in a textual corpus based on a cluster of words and phrases found in each document and their respective frequency. A document typically contains multiple topics in different proportions; thus, the widget also reports on the topic weight per document. In this analysis, the Latent Dirichlet Allocation (LDA) method was chosen for topic modeling. The most important model parameter, which has to be determined in advance, is the number of topics [57]. Based on our judgment and with the help of the Marginal Topic Probability (MTP) widget, only the first five topics (from 10 topics chosen by default) were selected as topics that best describe the content of the corpus. By combining the widget topic modeling with the corpus viewer and data table widget, the probability of the appearance of a certain topic in each paper was revealed, ranging from 0 to 1. This means that all papers assigned with a probability greater than zero can be related to a given topic. To explore the relationship between frequent and specific words in a certain topic, the LDA-based visualization (LDA-vis) widget was connected to the topic modeling widget, and top-ranked words for each topic, weighted by relevance, were disclosed. The relevance factor in the LDAvis widget was automatically set to 0.5.

**Figure 1:**
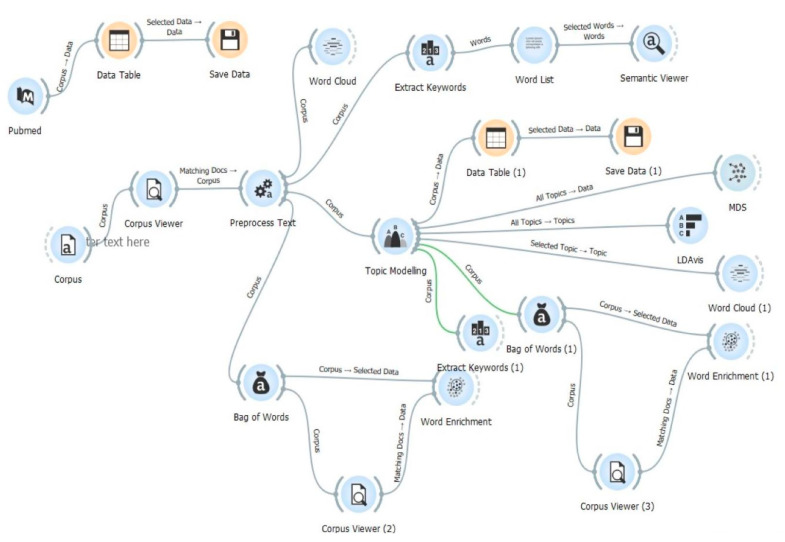
The workflow used for text mining analysis.

In Figure 1, the widgets employed for the text mining workflow are illustrated.

## 3. Results and Discussion

### 3.1. PubMed Search

Using the predefined search criteria, the PubMed widget found 4332 publications. Although the PubMed widget was set to extract only those papers published in the past decade, the widget extracted all papers that contained the assigned keywords since 1977. Due to this failure of the widget, the retrieved articles were manually screened. After excluding 1427 papers based on the year of publication, in total 2905 papers were included for further analysis.

**Figure 2:**
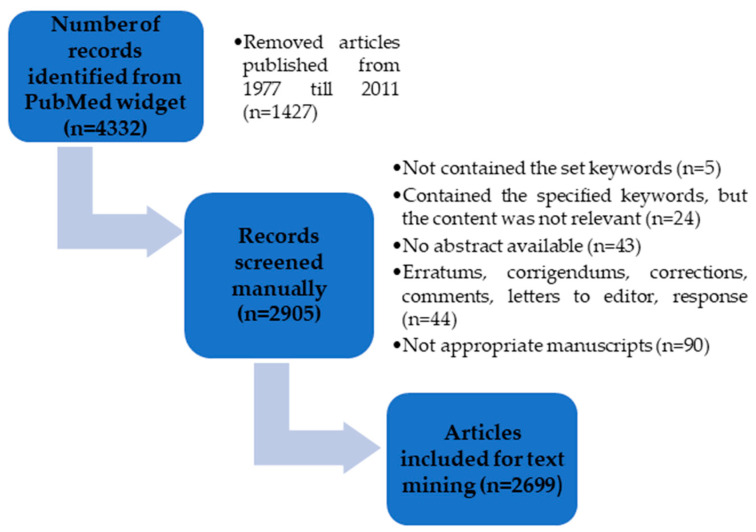
A summary of the papers considered at each stage of the review process and total number of extracted papers.

The selectivity of the PubMed widget towards the search criteria was rather good, since almost all of the extracted papers contained at least one of the set keywords either in the title, abstract or within the article’s keywords. The only exception was five articles that contained the keyword “PBBM” (meant to denote a phrase “physiologically based biopharmaceutics”) because this abbreviation is also used for other phrases such as “porous bovine bone mineral” or “phosphate-buffered basal media”. Further, those articles that did not satisfy the pre-set eligibility criteria (available abstract and appropriate manuscripts) were excluded, and in total 2699 papers were included for the final text mining analysis. Figure 2 summarizes the number of papers considered at each stage of the review process and final number of article abstracts extracted for text mining.

**Figure 3:**
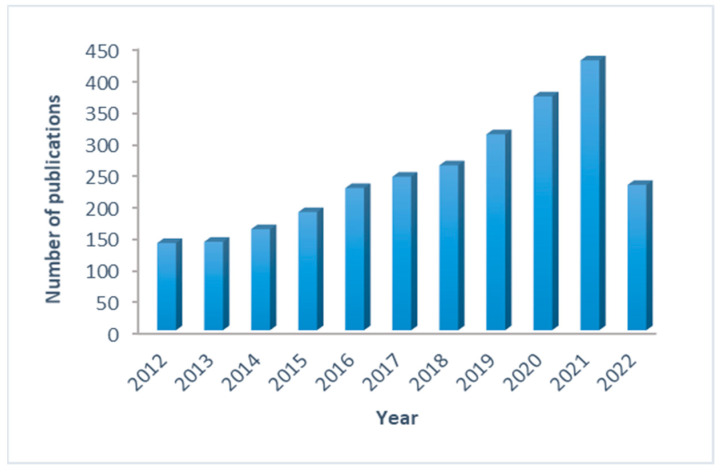
Number of publications on PBPK modeling per year.

Regarding the distribution of the selected publications per year (Figure 3), the number of papers published per year rose steadily with time, from 138 papers in 2012 to 427 papers published in 2021, representing a threefold increase. The observed drop in the number of publications in 2022 does not illustrate the real case because the analysis covered only the first 5 months of the year.

### 3.2. Text Preprocessing and Word Cloud Generation

**Figure 4:**
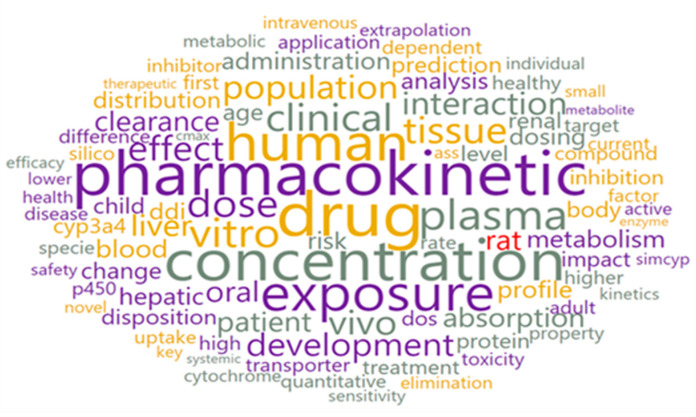
Word cloud for the whole corpus.

With the text preprocessing widget, 2699 text documents were converted into 74,635 tokens of 91 types. Using the word cloud option, all tokens were visually displayed (Figure 4).

The most frequently mentioned words in the 2699 analyzed abstracts are presented in Table 1. These data demonstrate that the obtained results are in accordance with the previously set search criteria. Additionally, the results from the words and weight list (Table 1) confirm the diverse application of PBPK modeling in different research areas.

**Figure 5:**
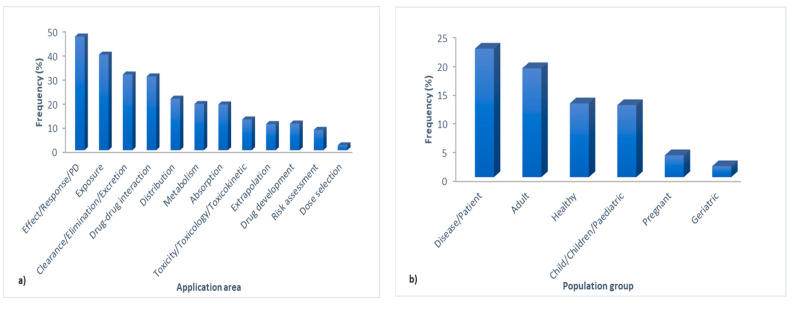

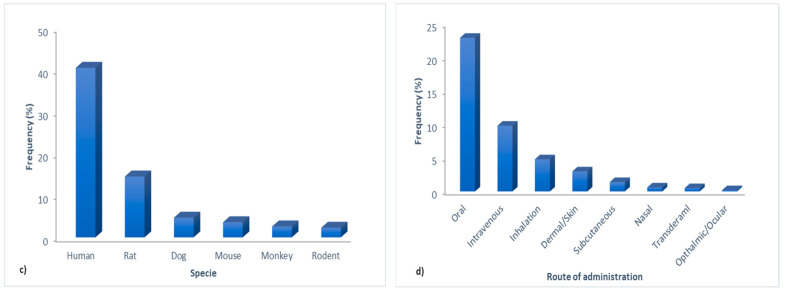
Distribution of PBPK modeling application: (**a**) across the analyzed publications; (**b**) for different population groups; (**c**) for different species; (**d**) for different administration routes.

Since the aim of this review was to investigate the versatility of PBPK modeling applications across retrieved publications, by using the extract keyword option and RegExp filter of the corpus viewer, the presence of certain words/phrases was measured. The obtained results (Figure 5a) revealed that the words “effect”/”response”/”pharmacodynamics” are the most frequently present across the published papers (47%) followed by the words “exposure” (39%) and “ddi/drug-drug interaction”(31%), while the phrase “dose selection” is the least frequently mentioned (2%). In relation to the distribution of the PBPK modeling application for different population (sub)groups, more frequent mentioning of the words “disease” and “patient” in comparison to the word “healthy” (Figure 5b) suggested that the majority of the analyzed papers dealt with PBPK modeling as a tool to predict drugs PK in different patient populations, e.g., in hepatic or renally impaired patients (4% for “renally” and 2% for “hepatic”). On the other hand, analysis of the distribution of PBPK modeling across different age groups revealed that the word “adult” had a higher word count than the words “children”/”child/”pediatric” (Figure 5b). The number of publications on PBPK modeling in the geriatric population and pregnant women is still rather scarce (Figure 5b), but this trend may likely change in future years [18]. Concerning the application of PBPK modeling for different species, the text mining results revealed that most of the published PBPK models referred to human representatives (40%), while a lesser proportion of papers described animal PBPK models, mostly rat models (15%) (Figure 5c). The distribution of PBPK modeling application regarding drugs administration routes was also evaluated, and the results showed that most of the published PBPK models were used to simulate oral drug administration (23%), followed by intravenous and inhalation administration (10 and 5%, respectively), while the ocular administration route has received only marginal attention so far (Figure 5d).

The analysis was extended to assess the proportion of published studies that include PBPK model validation. For this purpose, only original research articles were analyzed. Manual screening of the collected publications pool (2699 papers in total) retrieved 1929 original research articles. Incorporation of the words “validate” and “validation” in the RegExp filter of the corpus viewer widget revealed that, out of the 1929 original research articles, the selected words were present in only 232 articles. Such results indicate that only a small proportion of publications included the important step of PBPK model validation. The additional manual search of these 232 articles revealed that most of the published PBPK models were validated based on literature data (51%), and 26% were verified using experimental data. The analysis also revealed that 10% of the published models were validated using both literature and experimental data, while 13% of the extracted articles contained no information on the model validation procedure in the abstracts. Lastly, the original research articles were evaluated in relation to the used platform/software for model development. For this purpose, the selected keywords that represent common PBPK modeling software packages were incorporated into the RegExp filter of the corpus viewer widget. The results indicated that the word “simcyp” was mentioned in 11% of the analyzed articles, followed by the word “gastroplus” in 5% and “pk-sim” in 3% of the articles. Publications related to the use of other software such as MATLAB, Stella, NONMEN, WinNonlin, Berkeley Madonna and Simbiology were less frequent. The obtained results are in line with the review conducted by El-Khateeb et al., which indicated the prevalence of Simcyp platform in 55% of the published papers, followed by GastroPlus and PK-Sim [37]. It should also be noted that the majority of the original research articles analyzed in this review did not provide information on the used software in the article’s abstract.

### 3.3. Topic Modeling

By using the so-far described method, it was possible only to determine in how many papers a certain word and/or phrase appeared, without explaining the context in which it was used. Therefore, the topic modeling widget was used to identify specific topics within the retrieved abstracts.

The first five topics set by the LDA algorithm were considered as adequate representatives of the whole corpus based on the good values for log perplexity (16.7) and topic coherence (0.44) [58]. The 10 most common words that best describe each topic are shown in Table 2.

**Figure 6:**
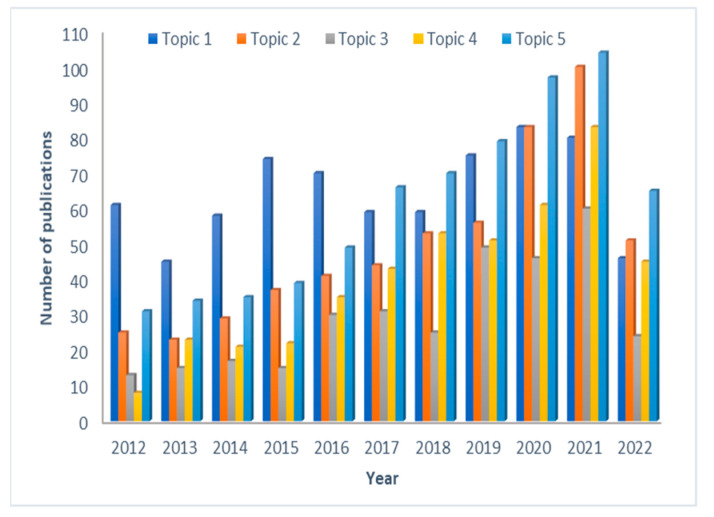
Growth in the number of published papers within each topic in the past 10 years.

Regarding the probability of occurrence of a certain topic in each publication, manual counting and screening of the retrieved articles revealed that 711 articles (26%) belong to topic 1, followed by topic 5 presented in 671 articles (25%), topic 2 in 496 articles (20%), topic 4 in 445 articles (17%) and topic 3 in 327 articles (12%). These results coincide with the results of the MTP value obtained with the MTP widget option. The growth in the number of publications within each topic during the past 10 years is shown in Figure 6. In the period between 2012 and 2017, mostly papers that belonged to topic 1 were published. However, starting from 2017 and onwards, a significant increase in the publications that belong to topic 5 can be observed. Therefore, in the last 3 years, there is a prevalence of published papers related to topic 5, which may be related to the fact that the leading regulatory authorities (EMA and FDA) have recognized the value of PBPK modeling in this area, and several PBPK modeling related guidances have been issued [39,40,59]. Similarly, publications related to topic 2 have constantly been increasing, and from 2020 the number of publications in this topic exceeded the number of publications related to topic 1. Starting from 2012 to 2021, increased interest in topic 4 and topic 3 can be seen through the cc, a ninefold increase for topic 4 and cc and a fourfold increase in topic 3. This is indicative of the growing maturity and specificity in PBPK applications across diverse fields.

#### 3.3.1. Topic 1—Early Drug Development, Risk Assessment and Toxicity Assessment

**Figure 7:**
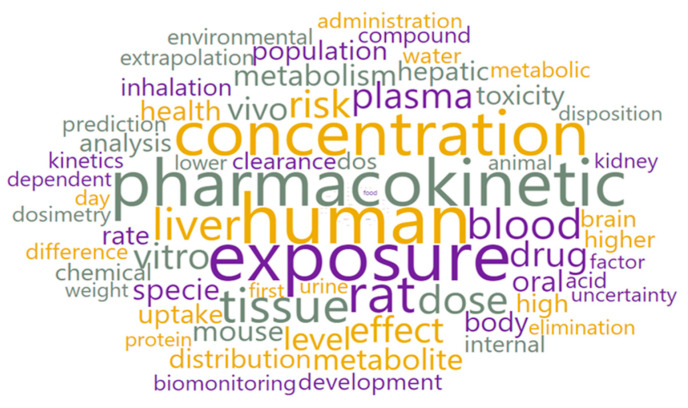
Word cloud for topic 1.

Figure 7 shows the representative words of topic 1 including “rat”, “distribution”, “toxicity”, “human”, “tissue”, “level”, “risk”, “chemical”, “liver”, “brain”, “blood”, “specie”. These words coincide with the statistically significant words extracted with bag of words and word enrichment widgets (Table S1). Statistically significant words for each topic are listed in Table S1 of the Supplementary Materials. The results of the LDAvis analysis matched the results obtained with the word cloud widget and word enrichment widget. Namely, the LDAvis analysis revealed that, when compared to the overall frequency, the words “rat”, “specie”, “health”, “human”, “toxicity” and “risk”, are present in 95, 95, 87, 78, 68, 68 and 62% of the articles that belong to topic 1, respectively.

These results suggest that topic 1 mainly refers to the utilization of PBPK modeling during early drug development, for the optimization and screening of promising compounds with desired/acceptable PK properties. Additionally, the words “extrapolation”, “rat” and “specie” imply that articles within topic 1 use PBPK in conjunction with in vitro- in vivo extrapolation (IVIVE) for the extrapolation of drug PK data across species. Furthermore, topic 1 clusters the articles that mention the PBPK modeling application for the assessment of human health risk associated with exposure to certain drugs/environment chemicals/mixtures of chemicals, as observed from the frequency of the words “health”, “risk” and “toxicity”.

**Figure 8:**
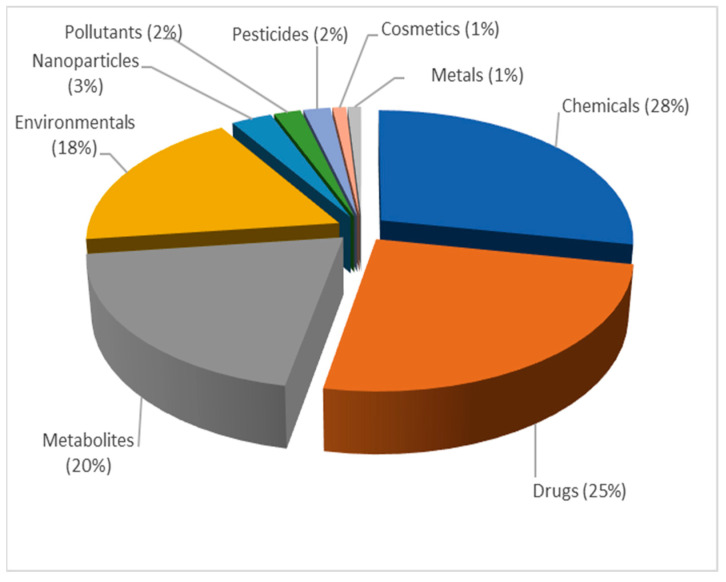
Distribution of published PBPK models for different types of compounds within topic 1, expressed as frequency (%), normalized to 100%.

In relation to the specific keywords observed within the word cloud for topic 1 (Figure 7), the extracted articles that belong to this topic were divided into several categories and further analyzed. First, the distribution of the published PBPK models for different types of compounds was investigated (Figure 8). Based on the representation of each word in the extracted publications, the results indicated that many of the published PBPK models were developed for “chemicals” (28%), followed by “drugs” (25%), “metabolite(s) of drugs/chemicals” (20%) and “environmental compounds” (18%). A much smaller proportions of PBPK models were developed for “nanoparticles” (3%), “pollutants” (2%), “pesticides” (2%), “cosmetics” (1%) and “metals” (1%).

Next, the distribution of PBPK models in relation to the prediction of human exposure to various compounds via different pathways was investigated. The results showed that the majority of the analyzed publications used PBPK modeling for the prediction of human exposure to drugs/chemicals following oral ingestion (25%), of which the majority represents ingestion through water (11%) and fewer ingestion through food (9%). Except for the oral route of administration, a large number of articles describe the use of PBPK modeling for the prediction of certain chemicals/pollutants/drugs exposure through inhalation (15%), whereas the intravenous and topical exposures are not frequently addressed. Regarding the application of PBPK modeling for species extrapolation, the majority of articles describe the use of human models, with the word “human” being more present in the extracted publications, followed by “rat” models.

As for the employment of PBPK modeling for the prediction of drugs/chemicals PK properties, the majority of articles mention PBPK models in terms of predicting drugs/chemicals blood/plasma concentration (56%), followed by the prediction of tissue concentration (37%) and urine concentration (only 11%). Furthermore, it was observed that the majority of publications within topic 1 describe the use of PBPK models for the prediction of drugs/chemicals excretion/clearance and metabolism (24%), followed by tissue distribution (23%). Since the words “silico” and “vitro” are present in 50% of the articles in topic 1, it can be presumed that the published PBPK models within topic 1 were generated mainly based on in silico and in vitro data as inputs.

The steady rise of published papers within topic 1 indicates that PBPK models are valuable tools, and their use in topic 1 related areas has been constantly increasing from 2012 till 2020 (Figure 6). Applying IVIVE in conjunction with PBPK modeling can support early drug discovery by considering the expected absorption, distribution, metabolism and excretion (ADME) processes in chemical/drug screening and providing mechanistic explanations of chemicals/drugs bioactivity observed in vitro [60]. The results of the text mining analysis are in compliance with the review article of Thompson et al., who systematically reviewed the published PBPK models and assessed their application across different types of compounds [44]. According to their review, PBPK models have been used to predict body exposure to a large number of compounds, including food additives, cosmetic ingredients, drugs, botanicals, pesticides etc. They also showed that, regarding the distribution of PBPK models application across species, the human models were predominantly present, followed by rat and mouse models.

#### 3.3.2. Topic 2—PBPK Modeling in Specific/Diseased Populations

**Figure 9:**
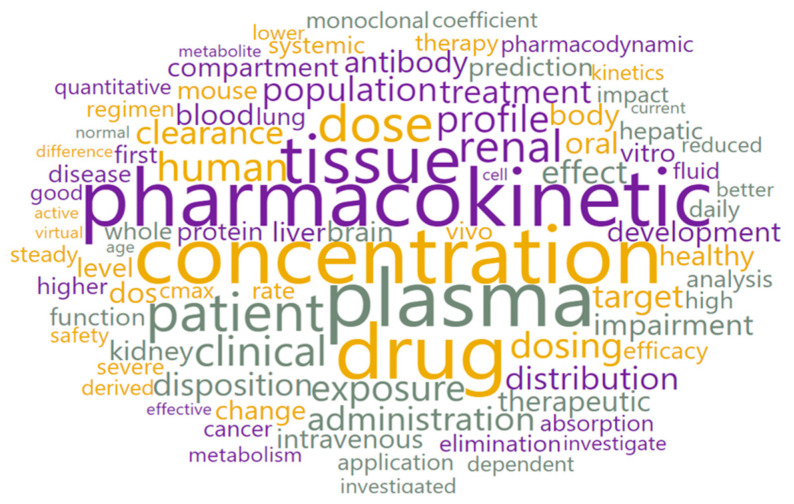
Word cloud for topic 2.

As for topic 2, the results of the word cloud (Figure 9) and word enrichment widgets (Table S1) revealed that the most frequently occurring and statistically significant words are “patient”, “plasma”, “treatment”, “dosing”, “dose”, “renal”, “efficacy”, “therapeutic”, “intravenous”, “disease”, “clinical”, “population”. Additionally, the results of the LDAvis analysis demonstrated that the words “renal”, “patient”, “plasma” and “intravenous” are present in 88, 77, 67 and 59% within topic 2, respectively.

**Figure 10:**
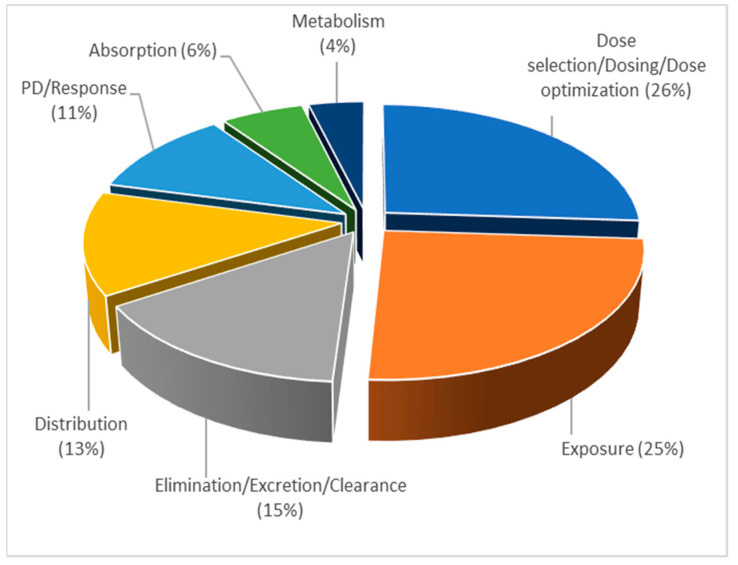
Distribution of published PBPK modeling per application area within topic 2, expressed as frequency (%), normalized to 100%.

Thus, topic 2 comprises the articles that explain utilization of PBPK modeling in the early clinical development, as a tool to extrapolate or predict PK changes in specific populations, including various disease groups. In the articles within topic 2, PBPK models were mainly used for dose optimization in different populations (26%) and prediction of a drug’s exposure (25%), as seen in Figure 10.

**Figure 11:**
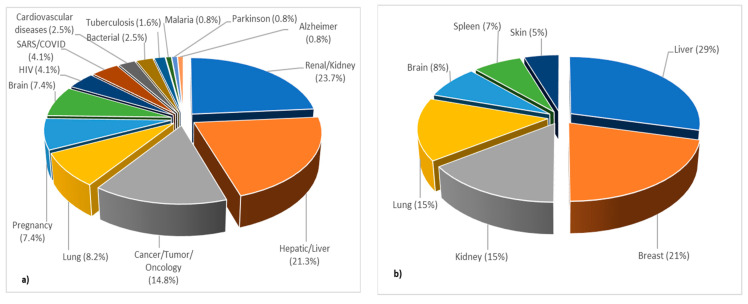
Distribution of PBPK modeling application: (**a**) per disease type; (**b**) per type of cancer within topic 2, expressed as frequency (%), normalized to 100%.

The published papers belonging to topic 2 were further divided and analyzed in relation to the distribution of the described PBPK models for different types of diseases (Figure 11). The RegExp filter and extract keyword widget showed that in 24% of the publications, PBPK models were developed to predict drug disposition in renally impaired patients, while 21% of the published models were developed for prediction of drug disposition in patients with hepatic impairment. The analysis also showed that in the extracted articles PBPK modeling was used to predict drug responses for bacterial and viral (e.g., HIV, Covid-19) infections, brain disorders (such as Parkinson, Alzheimer) and cardiovascular diseases (heart failure, thrombosis) but to a much lesser extent (Figure 11a). Further, by measuring the presence of the words “cancer”, “tumor” and “oncology”, it was revealed that 16% of the published papers in topic 2 used PBPK modeling to predict the response or disposition of a drug in patients with cancer. Of those 16%, most of the published PBPK models refer to liver cancer (29%) and only 5% to skin cancer (Figure 11b).

PBPK modeling is especially useful for prediction of drug-disease-related interactions because this approach can incorporate the multitude of pathophysiological changes in organ impairment and their impact on drug bioperformance. In this way, PBPK models can predict the impact of disease on the exposure of drugs that are either renally or hepatically eliminated [61]. With the help of PBPK models, the number of clinical trials needed in dose-finding studies in a specific population can be reduced. As seen in Figure 6, the number of publications that utilized PBPK modeling to support model-informed drug dosing in chronic conditions is constantly increasing. This is in line with the fact that both EMA and FDA suggest using PBPK modeling and simulation to increase the confidence of the study design and trial findings in these specific populations [62,63,64].

#### 3.3.3. Topic 3—Pediatric PBPK (P-PBPK) Modeling

**Figure 12:**
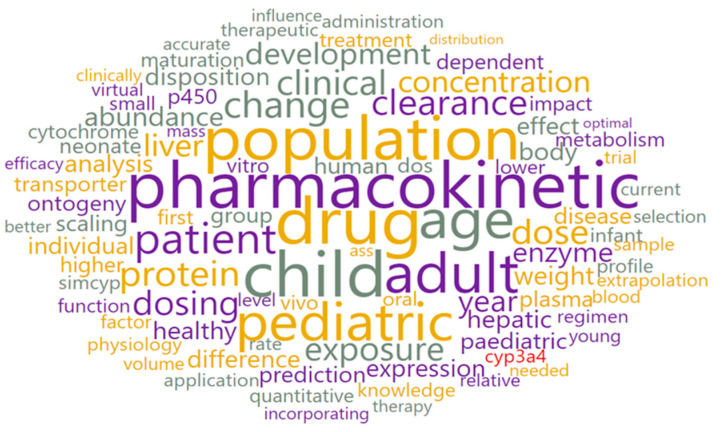
Word cloud for topic 3.

The presence (Figure 12) and significance of the words (Table S1) such as “child”, “pediatric/pediatric”, “population” and “age” suggest that the articles within topic 3 address the use of PBPK modeling in pediatric drug development. With the aid of the LDAvis analysis widget, it was confirmed that, when compared to the overall frequency, the words “child”, “age”, “adult” and “population” are dominantly present in articles within topic 3 (100, 98, 95 and 62%, respectively).

**Figure 13:**
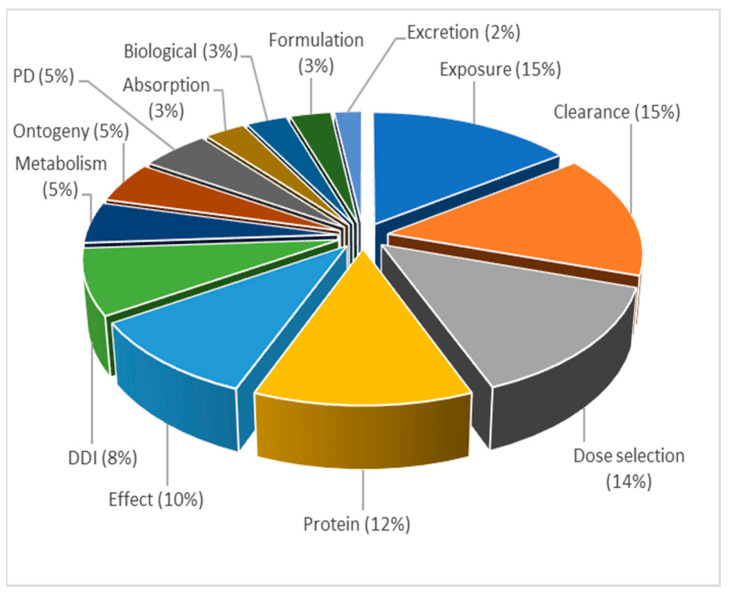
Distribution of published P-PBPK models per application area within topic 3, expressed as frequency (%), normalized to 100%.

Since P-PBPK models are widely used in pediatric drug development, the extracted articles within topic 3 were further analyzed in terms of application of these models in different areas (Figure 13). The results revealed that the words “exposure” and “clearance” were present in 15% of the articles, followed by the word “dosing” (14%). In contrast to this, the words “ontogeny” and “maturation” were present in only 5% of the articles, which indicated that P-PBPK models were less frequently developed to inform enzyme ontogeny.

**Figure 14:**
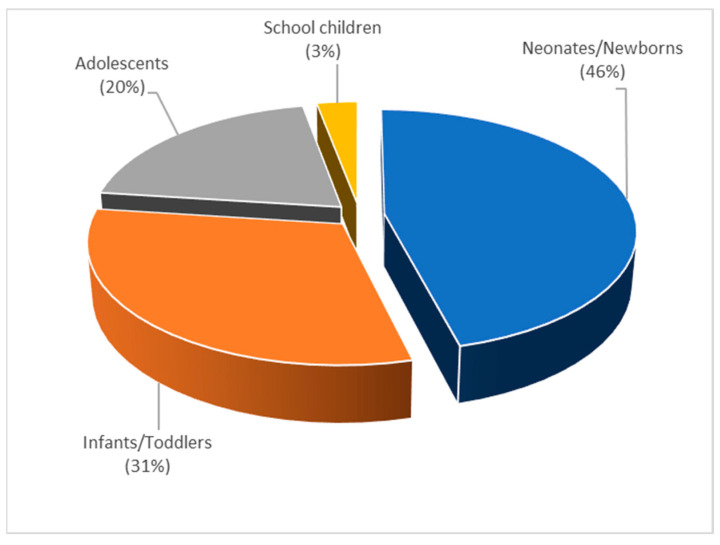
Distribution of published P-PBPK models within topic 3 for different pediatric age groups, expressed as frequency (%), normalized to 100%.

Regarding the distribution of P-PBPK modeling application through different age groups, most of the published P-PBPK were constructed for neonates, followed by toddlers and adolescents (Figure 14). Furthermore, in 45% of the publications the word “patient” was observed. By selecting the words “liver”, “hepatic”, “renal” and “kidney”, it was revealed that 49% of the published P-PBPK models were used to assess the impact of hepatic maturation on drugs’ PK in children and/or to make dose adjustments for drugs that are extensively metabolized in the liver. On the other hand, 29% of the published articles described the use of PBPK modeling to predict drugs’ bioperformance and make dose adjustments for renally cleared drugs in children.

PBPK modeling alone or in combination with population pharmacokinetics (popPK), has the potential of becoming an integral part of pediatric drug development. Since PBPK models incorporate the physiological and anatomical differences between adults and children, they offer the advantage of predicting a drug’s PK behavior in children and provide more reliable recommendations regarding pediatric dose adjustments when compared to dose calculations based on a child’s age, body weight and surface area. Within the literature, there are several research articles that confirm the benefit of pediatric PBPK models in different areas such as dose selection for children of different age, for optimization of clinical drug trials design, as well as for assessment of potential DDIs [65,66,67,68,69,70]. The importance of P-PBPK modeling in pediatric drug development is also emphasized in the review of Johson et al., who reported a 33-fold increase in the number of publications involving P-PBPK modeling from 2005 till 2020 [71]. The substantial role of PBPK modeling in pediatric drug development is also evident from the fact that these modeling tools are recommended in the FDA draft guidance on pediatric studies [72].

#### 3.3.4. Topic 4—Drug-Drug Interactions

**Figure 15:**
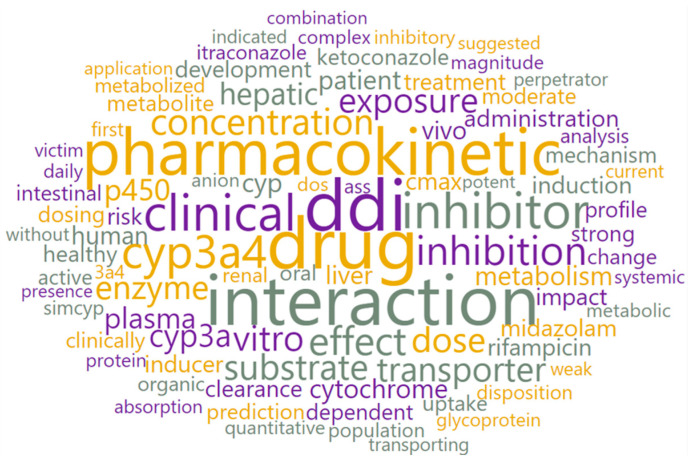
Word cloud for topic 4.

Figure 15 and Table S1 present the results from the word cloud widget and word enrichment widget for topic 4. Statistically significant words that best describe this topic are “interaction”, “ddi”, “p450”, “cyp3a4”, “cytochrome”, “inhibitor”, “substrate”, “induction”, and “clinical”. According to the results of the LDAvis analysis, the words “ddi”, “cyp3a4”, “inhibitor”, “inhibition” and “interaction” are present in 99, 95, 92, 90 and 84% of the articles that belong to topic 4, respectively.

**Figure 16:**
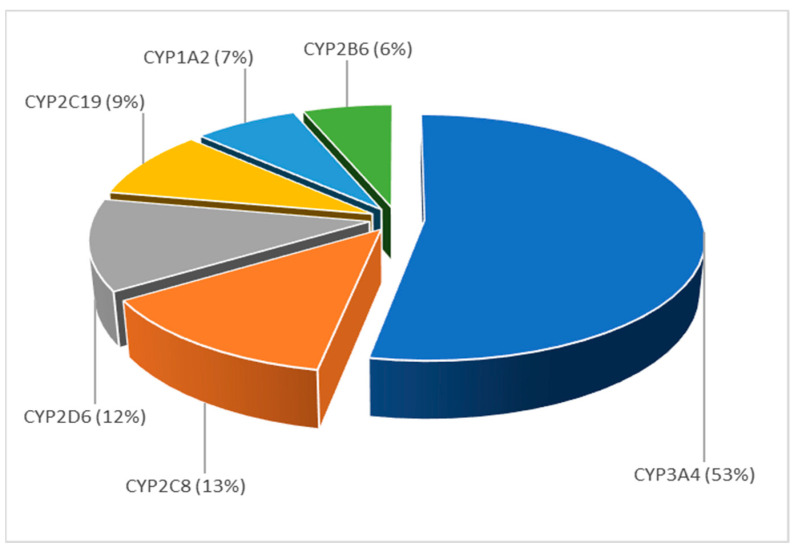
Distribution of published PBPK models in relation to the key analyzed CYP 450 enzyme within topic 4, expressed as frequency (%), normalized to 100%.

Such results indicate that the articles within topic 4 mostly describe the application of PBPK modeling to predict the potential for drug-drug interactions. The extracted keywords showed that published PBPK models were used to predict both enzyme- and transporter-mediated DDIs, with the word “enzyme” being more present (36%) than “transporter” (27%). Regarding the distribution of PBPK modeling in relation to the prediction of drugs’ ADME properties affected by DDIs, in the majority of published papers, these models were used to predict DDIs at the level of metabolism (30%), followed by absorption (12%), while only 7% of the models dealt with the prediction of DDIs at the level of drug distribution and elimination. By extracting the articles that contained the word “cyp”, and with the help of the extract keywords widget, the most frequent keywords within this subcorpus were analyzed. Figure 16 shows distribution of the published PBPK models in relation to the types of cytochrome (CYP) P-450 enzymes involved in drug metabolism and demonstrates that most of the published studies investigated the impact of CYP3A4-mediated DDIs. Additionally, our analysis revealed that 76% of the published PBPK models were used to predict DDI for investigational drugs that act as a weak or strong enzyme inhibitor, while 37% of the models assessed DDI when the investigational drug acted as a weak or strong inducer of CYP3A4 activity. A frequent presence of the words “ketoconazole”, “rifampicin”, “itraconazole” and “midazolam” within the articles in this topic implies that these model substances are generally used as inhibitors/inducers/substrates of various CYP enzymes to assess the potential DDIs. These data are in line with the EMA and FDA recommendations on probe substances for the in vitro and in vivo investigation of DDIs [73,74].

**Figure 17:**
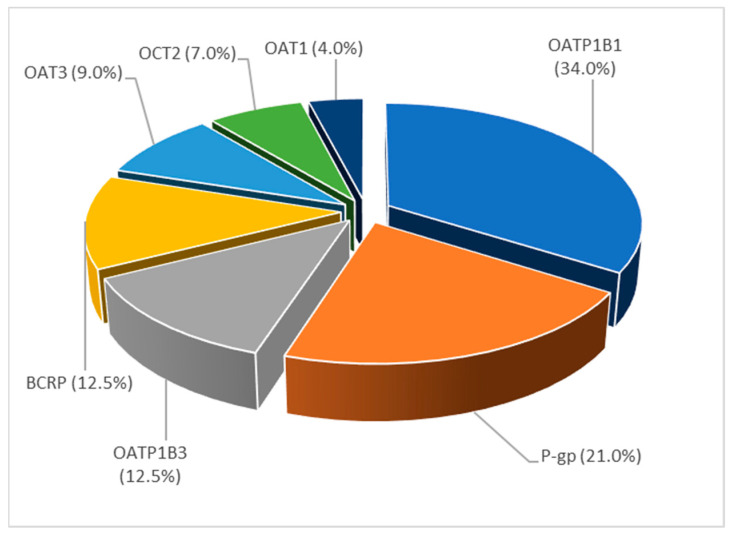
Distribution of published PBPK models in relation to the key analyzed transporter within topic 4, expressed as frequency (%), normalized to 100%.

Further, the utilization of PBPK modeling for the prediction of transporter-mediated DDIs was investigated, by extracting all publications that contained the word “transporter”. The extract keyword widget showed that frequently mentioned words within the subcorpus “transporter” are “intestinal”, “renal”, “liver”, and “clearance” indicating that most of the articles discuss utilization of PBPK modeling for the prediction of DDIs mediated by the transporters in the liver (32%), kidneys (20%) and intestine (17%). Figure 17 shows distribution of the published PBPK models in relation to the transporters involved in the simulated DDI. The results revealed that 34% of the published PBPK models were used to predict DDIs mediated by OATP1B1/1B3, followed by DDIs mediated by P-gp (21%). Furthermore, based on the higher presence of the words “inhibitor/inhibition” (76%) than the words “induction/inducer” (25%), it can be concluded that most of the studies within the subcorpus “transport” investigated the effect of inhibitors of transport proteins.

Concerning the use of PBPK modeling for the prediction of DDIs in special populations, the prevalence of the words “patient” (25%) and “healthy” (21%) within topic 4 indicates that disease-specific PBPK models are even more frequently used to estimate DDIs than the models for healthy representatives. Additionally, our analysis revealed that only 3% of the published PBPK models within topic 4 was used to predict DDIs in the pediatric population.

As seen from Figure 6, over the years there has been a cc. ninefold increase in the number of publications related to this topic. If appropriately validated and verified with clinical data, PBPK models may be used to simulate untested scenarios and support drug labeling [75]. Several research articles demonstrated the robustness in the predictive ability of the developed PBPK models, confirming the usefulness of these modeling tools for the assessment of DDIs [76,77,78]. Additionally, there has been an evident increase in the application of PBPK modeling for the assessment of DDI potential in regulatory submissions, which is supported by both EMA and FDA, who encourage sponsors to use in silico approaches to help translate in vitro observations into in vivo predictions of potential clinical DDIs [73,74,79].

#### 3.3.5. Topic 5—Drug Absorption Modeling and Physiologically Based Biopharmaceutics Modeling (PBBM)

**Figure 18:**
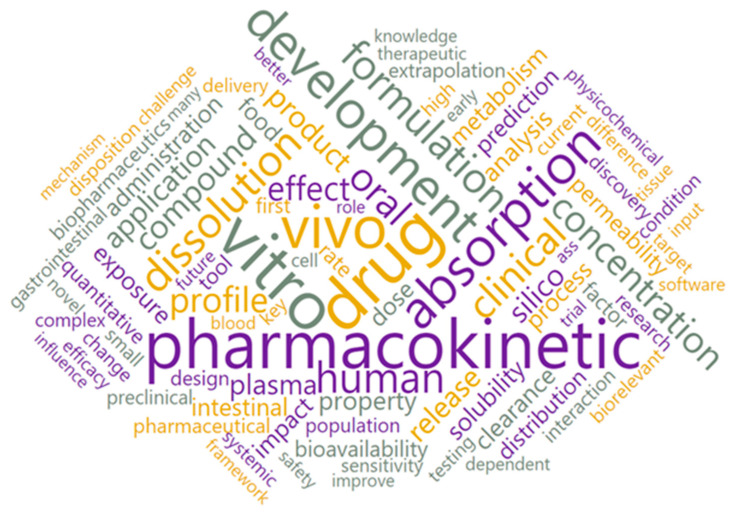
Word cloud for topic 5.

The word cloud (Figure 18) and word enrichment widgets (Table S1) revealed that statistically significant words within topic 5, which best describe the content of the topic are: “formulation”, “silico”, “dissolution”, “absorption”, “release”, “biopharmaceutics”, “permeability”, “biorelevant”, “food”, “gastrointestinal”, and “bioavailability”. Furthermore, according to the LDAvis analysis, the words “absorption”, “silico”, “vivo”, “vitro” and “development” are present in 89, 82, 79, 73 and 66% of the publications within topic 5.

**Figure 19:**
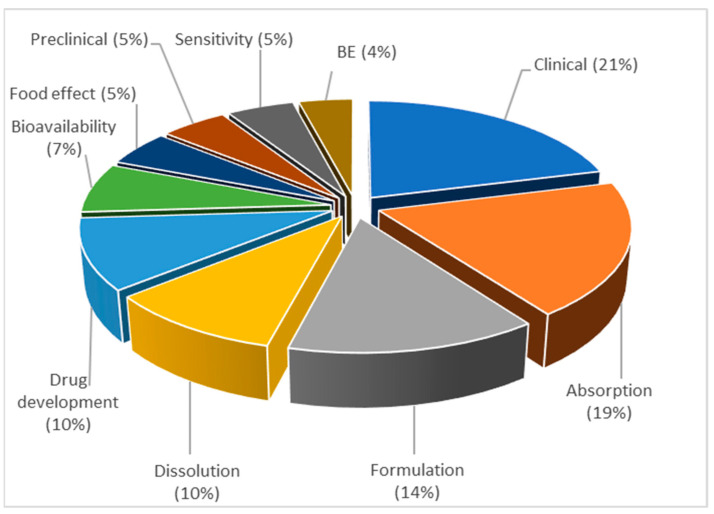
Distribution of published PBPK/PBBM models in relation to the application area within topic 5, expressed as frequency (%), normalized to 100%.

These results indicate that the articles within topic 5 describe the utilization of PBPK modeling tools during formulation development. In this context, PBPK modeling is often referred to as physiologically based biopharmaceutics modeling (PBBM) and denotes a link between biopredictive in vitro dissolution testing and mechanistic oral absorption modeling [80]. Because this topic covers a relatively wide application range, the distribution of the published PBPK models across different application fields was firstly explored. Figure 19 shows the distribution of the published PBPK/PBBM models in relation to the application area, defined by the representative keyword. As observed, the word “clinical” is the most frequently encountered within topic 5, present in 21% of the articles, which indicates that PBPK absorption models are mainly used to identify parameters affecting clinical drug performance or clinical outcomes for the tested drugs/formulations. The words “absorption”, “formulation”, and “dissolution” are also frequently mentioned in the publications within topic 5 (19, 14 and 10%, respectively), indicating that a number of articles within this topic addressed the use of PBPK/PBBM modeling in terms of assessing the impact of drug dissolution and other formulation properties on a drug’s absorption. On the other hand, only 4% of the articles contained the word “bioequivalence”, suggesting that these M&S tools have been only sparingly used during generic drug development to predict the outcome of a bioequivalence (BE) study.

After extracting the 290 articles that contained the word “absorption”, it was observed that the occurrence of the word “oral” is 58%. This is in line with the fact that most marketed drugs are administered orally, and therefore the main focus has been directed to develop PBPK models able to describe bioperformance of orally administered drugs [34]. Regarding the application of PBPK modeling tools for the prediction of drug absorption following non-oral administration routes, our results revealed that only a limited number of publications addressed the use of PBPK modeling for alternative drug dosing routes. In particular, 5% of the analyzed articles dealt with the inhalation drug dosing route, followed by dermal/topic (4%), subcutaneous (2%) and ocular (1%), and less than 1% for the nasal drug administration.

Sensitivity analysis (SA) is one of the key features of PBPK modeling, which is used to assess the sensitivity of the output variables (e.g., drug’s bioavailability and PK parameters describing the rate and extent of drug absorption) to the changes in the input variables (e.g., drug and formulation properties such as solubility, particle size, dissolution rate, permeability) [81]. Therefore, the articles that contained the word “sensitivity” were further evaluated to examine which input variables are most commonly subjected to SA. The results showed that most of the published studies (37%) used SA to identify the impact on drug dissolution from a formulation on the rate and extent of a drug’s absorption. Also, a notable portion of the published studies investigated the impact of a drug’s solubility (28%) and permeability (26%) on its absorption.

As already pointed out, PBPK/PBBM modeling has been increasingly utilized within academic and research institutions, the pharmaceutical industry and regulatory agencies (Figure 6). PBPK/PBBM models can be used for a diversity of applications, starting from the early stages of candidate selection, when these tools can facilitate the selection of molecule(s) with the best properties to attain sufficient absorption. Publications related to the utilization of PBPK/PBBM modeling as a tool to guide formulation development are even more numerous, and they report the use of SA on the influence of a drug’s and drug product’s properties on drug absorption [82,83,84]. Other advantages of PBPK/PBBM models include their potential to be used in the establishment of in vitro-in vivo correlation (IVIVC), waive future clinical studies [80,85,86]. Furthermore, the use of PBPK models for non-oral routes of drugs administration is expected to grow considering the initiatives of the FDA to advance the development of PBPK/PBBM modeling tools for dosage forms administered via, e.g., dermal, ocular and intraoral administration [87,88,89,90,91,92].

In the last step, estimation of the proportion of original research articles that include PBPK model validation per topic was performed. The analysis showed that the highest percentage of articles that include model validation belongs to topic 2 (37%), followed by topic 1 (26%), topic 4 (20%), and lastly topic 3 and topic 5 with only 9% of such articles. The highest percentage of experimentally validated models was included in the publications within topic 1 (44%), followed by topic 5 (32%), topic 2 (19%), topic 4 (15%) and lastly topic 3 (14%). On the other hand, the largest percentage of the models validated based on literature data was included in the publications that belong to topic 4 (60%), followed by topic 2 (58%), topic 3 (54%), topic 5 (45%) and topic 1 (33%). Such results may seem unexpected considering the fact that topic 1 deals with the utilization of PBPK modeling in the early drug discovery phase when only limited data on drug performance are available. However, it should be emphasized that topic 1 also includes publications related to the use of PBPK modeling for toxicological predictions, environment risk assessment and interspecies scaling, and these publications contributed to the large percentage of experimentally validated models within topic 1.

## 4. Limitations of the Applied Analysis

By applying several text mining techniques on a large sample of publications (2699), we managed to extract useful information on the application of PBPK modeling tools in recent years. However, we need to highlight certain limitations of the conducted review analysis. As already pointed out, the software’s integrated PubMed widget failed to extract the articles based on the period of publication, and therefore this task was performed manually. Another annotation concerns the topic modeling widget and its inability to ideally separate the publications within the defined topics. Namely, the widget only extracts the data on the percentage probability that a publication belongs to a certain topic, so one publication may belong to different topics, and this may complicate the overall analysis. Furthermore, regarding the analysis on the mention of model validation in the original research articles, as well as on the utilization of different software tools for PBPK modeling, the obtained results may not represent the actual number/proportion of the relevant publications since the text mining analysis was limited to the articles’ abstracts. Namely, the abstracts rarely mentioned the information of interest for this review. Additionally, we need to note that validity of the obtained results depended on appropriate pre-processing of the text and correct choice of stop words, as well as proper interpretation and linking of the results, which is supported by the authors’ domain knowledge on the studied topic.

## 5. Conclusions

This paper represents a successful application of text mining tools for thorough review, systematization and interpretation of research trends in PBPK modeling applications. The increasing number of PBPK-related publications over the years demonstrates the evident shift towards wider utilization of PBPK modeling tools in various research fields. The topic modeling analysis confirmed the application of PBPK modeling in diverse areas. The larger proportion of papers published in the last 5 years is related to the application of PBPK/PBBM modeling in formulation development. However, the number of publications related to other topics, such as modeling drug bioperformance in specific or diseased populations (including pediatric population), modeling of drug-drug interactions and modeling of drug properties (including risk assessment and toxicokinetic assessment) during early drug development is also constantly increasing. Although several reviews on PBPK modeling have been published in the past years, covering various modeling aspects, they generally indicate that PBPK modeling has been most commonly used to assess DDI and guide clinical DDI studies [37,41,44]. The results of this review are somewhat different, and the obtained results can partly be attributed to the differences in the eligibility criteria regarding the analyzed publications. Namely, previously published reviews focused on original research articles and the application of PBPK modeling in the assessment of a drug’s bioperformance (e.g., DDI predictions, pharmacokinetic predictions in special populations, predictions of drug absorption, selection of dosing schedule, assessment of inter-individual variations) using human models. [37,41,43,47,49]. On the other hand, the aim of this review was to investigate the utilization of PBPK modeling in pharmaceutical research and development, and also in related areas such as toxicology and environmental risk assessment, in which PBPK modeling has been well established [44]. For example, Feras and Laer published a review stating that although PBPK was originally generated within the pharmaceutic industry, it has become a commonly and widely used technique in the area of environment risk assessment and toxicology [46]. Additionally, a review conducted by Edginton et al. showed that 60% of all PBPK-related publications in the period 1980 to 2007 referred to the field of environment toxicology [93]. The use of PBPK models has changed the way that the pharmaceutical industry develops drugs, as well as the way regulators accept them. The ability to predict ADME characteristics of a drug in the early drug discovery phase and the expected drug’s PK profile and thus pharmacodynamic response in different patient subgroups could drastically reduce the time and costs of the overall drug and formulation development processes. However, the main challenge resides in the accuracy of PBPK modeling predictions. Model validation and verification are the key steps to increase confidence in the generated models and associated predictions. As shown in this review and previously published papers [41,43], a relatively small proportion of the published studies experimentally validated the applied PBPK models and reported criteria for model qualification. As a future perspective, a rigorous qualification concept should be established for PBPK models to ensure the validity of PBPK predictions.

## Figures and Tables

**Table 1:** Words & Weight list for whole corpus.

Word	Weight
Drug	6108
Pharmacokinetic	3552
Concentration	3251
Exposure	2642
Human	2567
Plasma	2094
Vitro	1892
Clinical	1785
Dose	1778
Tissue	1697
Effect	1482
Vivo	1429
Population	1347
Development	1196
Oral	1079
Interaction	1067
Clearance	1027
Patient	1016
Rat	949
Absorption	944
DDI	821
Metabolism	798
Dosing	821
Risk	738
Age	738
Distribution	711
Child	675

**Table 2:** Most common words within each topic (listed by descending frequency).

Topic 1	Topic 2	Topic 3	Topic 4	Topic 5
Human	Concentration	Population	Drug	Drug
Exposure	Plasma	Age	DDI	Vitro
Concentration	Tissue	Child	Interaction	Vivo
Rat	Pharmacokinetic	Drug	Clinical	Absorption
Liver	Dose	Pharmacokinetic	Pharmacokinetic	Development
Blood	Renal	Adult	Cyp3A4	Pharmacokinetic
Pharmacokinetic	Drug	Protein	Inhibition	Clinical
Tissue	Patient	Change	Exposure	Oral
Risk	Dosing	Dosing	Effect	Silico
Specie	Clinical	Clearance	Inhibitor	Effect

## Data Availability

Data are contained within the article.

## Supplementary Materials

The following supporting information can be downloaded at: https://www.mdpi.com/article/10.3390/pharmaceutics15010107/s1, Table S1: Statistically significant words for each topic extracted with bag of words and word enrichment widgets.
