# Supplementary material for: In-Depth Analysis of Physiologically Based Pharmacokinetic (PBPK) Modeling Utilization in Different Application Fields Using Text Mining Tools
Source: Pharmaceutics. 2022 Dec 28;15(1):107. doi: 10.3390/pharmaceutics15010107 (PMC9860979; doi:10.3390/pharmaceutics15010107)
Supplement: Supplementary file 1 [file pharmaceutics-15-00107-s001.zip › pharmaceutics-2082938-supplementary.pdf]

Electronic Supplementary Material

Supplement S1: Summary of statistically significant words for each topic extracted with bag of words and word enrichment widgets

Table S1. Statistically significant words for each topic.

	Word	p-value
Topic 1	Rat	1.7×10^{-55}
	Human	2.7×10^{-47}
	Health	4.7×10^{-43}
	Exposure	5.3×10^{-36}
	Risk	6.7×10^{-36}
	Blood	3.0×10^{-32}
	Specie	2.8×10^{-22}
	Tissue	9.6×10^{-18}
	Liver	2.2×10^{-16}
	Toxicity	7.4×10^{-14}
	Extrapolation	1.8×10^{-3}
	Distribution	2.5×10^{-3}
Topic 2	Plasma	5.0×10^{-42}
	Patient	5.6×10^{-41}
	Treatment	5.7×10^{-38}
	Dosing	4.0×10^{-29}
	Renal	2.5×10^{-16}
	Efficacy	3.2×10^{-12}
	Therapeutic	4.7×10^{-12}
	Intravenous	9.2×10^{-11}
	Healthy	1.5×10^{-9}
	Clinical	8.0×10^{-8}
	Disease	2.0×10^{-6}
	Population	3.1×10^{-6}
Topic 3	Child	1.3×10^{-30}
	Age	2.9×10^{-29}
	Population	4.2×10^{-24}
	Adult	8.3×10^{-22}
	Dosing	7.4×10^{-9}
	Protein	1.9×10^{-7}
	Patient	1.2×10^{-5}
	Enzyme	8.2×10^{-5}
	Clearance	1.5×10^{-4}
	Clinical	1.4×10^{-3}
	Healthy	$2.5 \times 10\text{-}^{3}$
	Disease	$4.9 \times 10\text{-}^{3}$
Topic 4	Interaction	5.0×10^{-9}
	DDI	9.7×10^{-8}
	P450	4.2×10^{-6}
	Cyp3a4	9.0×10^{-6}

	Cytochrome	2.5×10^{-5}
	Inhibitor	3.4×10^{-5}
	Midazolam	3.1×10^{-4}
	Rifampicin	4.3×10^{-4}
	Induction	2.1×10^{-3}
	Weak	2.1×10^{-3}
	Moderate	2.7×10^{-3}
	Potent	9.5×10^{-3}
Topic 5	Formulation	5.4×10^{-5}
	Silico	1.0×10^{-4}
	Dissolution	2.5×10^{-4}
	Absorption	5.5×10^{-4}
	Release	3.9×10^{-3}
	Biopharmaceutics	8.6×10^{-3}
	Permeability	1.47×10^{-2}
	Biorelevant	1.7×10^{-2}
	Food	3.2×10^{-2}
	Gastrointestinal	4.2×10^{-2}
	Testing	4.5×10^{-2}
	Bioavailability	4.7×10^{-2}
